# Impact of Contraception on Uterine Fibroids

**DOI:** 10.3390/medicina57070717

**Published:** 2021-07-16

**Authors:** Katarzyna Kwas, Aleksandra Nowakowska, Angelika Fornalczyk, Magda Krzycka, Anna Nowak, Jacek Wilczyński, Maria Szubert

**Affiliations:** Clinic of Surgical and Oncologic Gynecology, 1st Department of Gynecology and Obstetrics, Medical University of Lodz, M. Pirogow Teaching Hospital, Wilenska 37 St., 94-029 Lodz, Poland; katarzyna.kwas@stud.umed.lodz.pl (K.K.); aleksandra.nowakowska@stud.umed.lodz.pl (A.N.); angelika.fornalczyk@stud.umed.lodz.pl (A.F.); madoka55@gmail.com (M.K.); annanowak27@wp.pl (A.N.); jrwil@post.pl (J.W.)

**Keywords:** contraception, oral contraceptive pills, uterine fibroids

## Abstract

*Background and Objectives*: Uterine fibroids develop in 25–40% of women of childbearing age; however, there are discrepancies resulting from population and socioeconomic differences. The pathogenesis of fibroids is not clear. The aim of the study was to assess the potential connection between the use of oral contraceptives and the occurrence of uterine fibroids in women of childbearing age. *Materials and Methods*: In this prospective, survey, case–control study, data were collected from Caucasian female patients (mean age = 30) using a questionnaire concerning the onset, duration and form of hormonal contraception, and medical and obstetrical history. The questionnaires were handed personally to hospitalized patients as well as distributed through Google forms on social media. *Results*: In a study group (*n* = 140) of patients using hormonal contraception, 37.8% of them were diagnosed with uterine fibroids, whereas among the patients not using hormonal contraception (*n* = 206), uterine fibroids were diagnosed in 59.6% of the patients. The most common hormonal contraception was two-component hormonal tablets used by 93.3% of the patients. Taking contraceptives was a uterine fibroids protective factor (OR = 0.4, *p* = 0.007). In the study group, 5.5% of the patients were pregnant and 60.42% were diagnosed with uterine fibroids (OR = 4.4, *p* < 0.000001). *Conclusion*: Contraception was found to be a protective factor for uterine fibroids among the women surveyed. The presented data confirm the theory about the hormonal dependence of uterine fibroids.

## 1. Introduction

Uterine fibroids are one of the most common, benign neoplasms affecting the reproductive tract in women. The most popular classification of these tumors is according to their location. The symptoms and treatment options are affected by the size, number and location of the fibroids [1]. Fibroids could be single or multiple and may vary in size. Their growth is estrogen-dependent because they have more estrogen receptors than the surrounding tissue [2]. It has also been proven that progesterone plays an important role in their pathogenesis, participating in their growth stimulation. It is estimated that uterine fibroids are the most common benign neoplasm occurring among menopausal women—this disease can affect over 70% of white women and more than 80% of Afro-American women during their lifetime, but this rate vary according to way of gathering epidemiological data [3]. Many women have an asymptomatic course of the disease; however, in about 25 to 50% of patients, clinical manifestations such as abnormal uterine bleeding, anemia, pelvic pain and pressure, back pain, urinary frequency, constipation or infertility have been reported. It is known that the incidence of fibroids increases with age until reaching menopause, followed by a downward trend. Their diagnosis is often made when controlling a woman using contraception. Contraception is also frequently used to reduce the symptoms caused by the excessive bleeding accompanying the fibroids. The main mechanism of hormonal contraceptive is to block ovulation through an antigonadotropic effect. The oral contraceptive pill (OC) is the most commonly used form of reversible contraception [4]. Most hormones used for contraception also contain estrogens or substances that are estrogenic. The gestagenic component impairs the luteal phase of the ovulatory cycle and changes the density of the cervical mucus, which hinders the movement of sperm. The estrogen component stimulates FSH (follicle stimulationg hormone) suppression, which, in turn, inhibits the growth of the follicle and also enhances the effect of progesterone and, together with it, hinders the implantation of an embryo by changing the endometrium in the secretory phase. Nevertheless, some older epidemiological studies recognize oral contraception as a protective factor against the appearance of uterine fibroids, but the data are inconsistent between the studied populations, probably because of the different availability of OC doses from the introduction of OC in the sixties of XX century until now [5,6]. Therefore, there is a need to further studies in this area. The aim of the study was to assess the potential connection between the use of oral contraceptives and the occurrence of uterine fibroids in women of childbearing age.

## 2. Materials and Methods

The case–control study covered the population of patients hospitalized at the M. Madurowicz Hospital in Łódź in the Department of Surgical and Oncologic Gynecology due to uterine fibroids (for diagnostic or treatment reasons) and the population of patients from social groups found on popular social media. The survey was collected from January to September 2020. A total of 314 patients took part in the study online, whereas 32 females were asked to fill in the survey during their hospitalization. Due to the pandemic situation and the restrictions in planned surgeries, the hospitalization of patients with myomas was only because of emergency reasons, mainly pain or bleeding. Hospitalized patients filled in the questionnaires personally on paper. Patients from social media were recruited regarding where they live and filled in questionnaires using Google forms. Questionnaires were the same for inpatient and outpatient respondents. A total of 349 women completed a questionnaire consisting of 30 questions about the presence of uterine fibroids and taking contraceptive drugs. Patients who had gone through the menopause (*n* = 3) were excluded from further analysis. All respondents gave their consent in writing to participate in the study. Online respondents gave their consent by ticking the appropriate box after the informed consent statement. There was no possibility to answer the online questionnaire without previous consent. Patients were divided into a study group of 140 women with uterine leiomyomas and a control group without any lesions (206) based on an ultrasound examination.

All ultrasound scans obtained during the hospitalization of patients at the Madurowicz Hospital were performed using Mindray DC-70(Shenzhen Mindray Bio-Medical Electronics Co., Ltd. Shenzhen, China) with transvaginal and convex probes. Those scans were performed in a longitudinal and transversal plane. For each lesion, the two largest diameters were measured, and the precise location (anterior, posterior or lateral) was reported. All lesions were classified as leyomiomas according to the FIGO classification system (PALM-COEIN). To verify the fibroids presence in online respondents, the questions about ultrasound diagnosis had to be answered before proceeding to the next part of the survey. Patients were asked about the date of the diagnosis, where and by whom the ultrasound was performed and what the final result was (submucosal myoma, intramural, subserosal, others). The data regarding the place of the diagnosis and location of the myomas were not further statistically analyzed due to high heterogeneity of examiners (all over the country). The approval of the Bioethics Committee No. RNN12/20/KE was obtained for the study. Statistical analysis was performed using Statistica Programme version 13.5 using U-Mann–Whitney test and Chi2 test for non-parametric variables with a *p* value < 0.05 as statistically significant.

## 3. Results

Generally, 346 females took part in the study, with 140 females in the study group and 206 in the control group, respectively. A total of 146 respondents were pregnant before the study; among these, 60.7% had uterine fibroids. In the group of females who were not pregnant, 25.6% had uterine fibroids. The data concerning the characteristics of the studied groups are presented in Table 1.

### 3.1. Contraception

Contraception was used by 274 patients, which was 79% of all the participants, and 34.3% of them had uterine fibroids. In the group of patients who did not use contraception, 63.4% of them had uterine fibroids. After the Chi-squared test analysis, the relationship between contraceptive use and the lack of uterine fibroids was statistically significant (*p* < 0.001). The mean age at which women began to use contraception was equal to 21 years (SD = 5.6) for the whole group of all the patients. The mean duration of contraceptive use was 51.2 months, SD = 59.5. The particular data concerning contraceptive use by the patients is presented in Table 2.

To exclude the possible influence of age on the development of myomas, a multivariate analysis was performed. For statistical purposes, the women were divided into the following age groups: 20–30, 30–40 and 40–50, with the assumption that contraception intake was longer than one year (to avoid possible bias in the youngest group, where the percentage of short-term users was the highest). The patients who reported having myomas before they began to use contraception were also excluded from further analysis. The impact of contraception on the occurrence of myomas were not visible in the youngest and oldest group of patients. Contraception served as a protective factor for uterine fibroids in the group of 30–40 years (Table 3).

Further analysis showed that 24.8% of all the patients had taken the morning-after pill at least once. In 38.4% of them, uterine fibroids were identified. On the other hand, in the group of patients who had not taken the morning-after pill, 40.9% had uterine fibroids. The Chi-squared test shows that there is no statistically significant relationship between morning-after pill use and uterine myomas presence *p* = 0.67559. The mean age of the first morning-after pill use was 22.3 years, SD = 5.7 (24.1 SD = 7.3 in the study group vs. 21.2 SD = 4.2; *p* = 0.07 in the control group).

**Figure 1:**
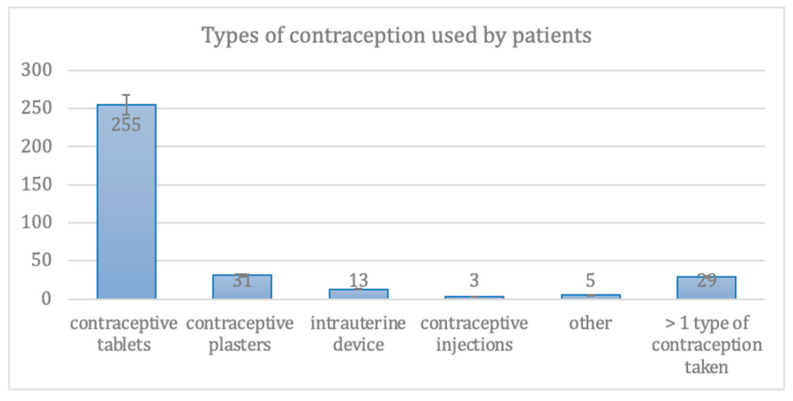
Types of contraception used in the group of patients who use contraceptives.

In the group of patients who used contraceptives, 84.5% of them took tablets, 9.3% used contraceptive plasters, 3.7% used intrauterine devices, 0.9% used contraceptive injections and 1.6% used other types of contraceptives. No statistically significant relationship was observed. The data are presented in Figure 1.

### 3.2. Medical-History

**Figure 2:**
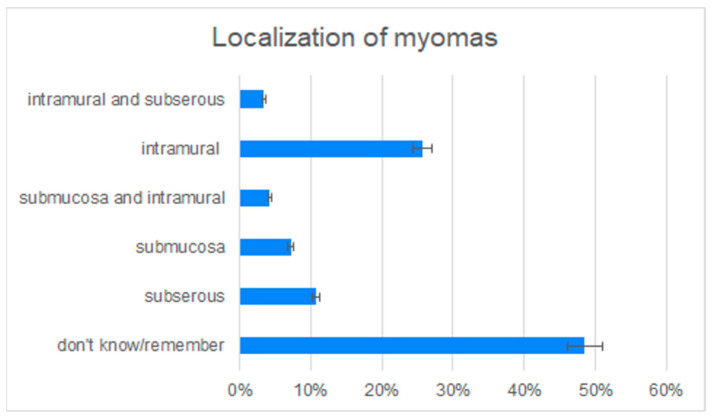
Localization of the myoma reported by the patients.

A total of 95 patients (27.4% of all patients) had a positive family history regarding uterine myomas; 57.9% of them had uterine fibroids either. In the group of patients without a positive family history, only 33.6% presented with uterine fibroids (Chi–Pearson test, *p* < 0.001). The patients using contraception with a positive family history were less often diagnosed with uterine myomas (*p* < 0.001; two-way ANOVA test). The mean age of menarche was 12.8 years, SD = 1.8 (13, SD = 2.1 in study group vs. 12.7, SD = 1.6 in control group). The relationship between the age of menarche and uterine fibroids diagnosis was not statistically significant between the groups (*p* = 0.2298). Gynecological surgeries were performed in 126 of the patients, among them 70.6% had uterine fibroids, whereas only 22.8% patients were diagnosed with uterine fibroids without previous gynecological surgeries. The Chi2–Pearson test shows a strong dependence between the identification of uterine fibroids and performed gynecological surgeries, such as hysteroscopy or hysterosalpingography, *p* < 0.001. Coexisting gynecological diseases (such as endometriosis, ovarian cysts) were stated in 24.6% of participants, 54.1% of them were also diagnosed with uterine fibroids; a statistically significant relationship between the presence of uterine fibroids and coexisting gynecological diseases was observed *p* < 0.001 (Chi2–Pearson). Of the healthy patients without coexisting gynecological diseases, 35.8% were identified with uterine fibroids. Hypertension was stated in 4.5% patients and 66.7% of them were diagnosed with uterine fibroids, whereas only 39.1% of patients without hypertension had uterine fibroids (*p* = 0.05593). A total of 140 patients (40.4% of all the patients) had uterine fibroids diagnosed. The mean age of patients’ uterine fibroids diagnosis was 32 years, SD = 7.3. Nearly half of the patients could not remember the localization of the myoma, the others reported locations, as shown in Figure 2.

All of the relationships analyzed in this study are present in Table 4.

## 4. Discussion

In our study, we show that those who have not already had uterine fibroids may have a smaller chance of developing them when taking contraceptive medications, especially pills that contain low-dose estrogens. The positive protective effect of OC has been particularly seen in the group of 30–40-year-old patients. The lack of the same protective effect in the older group of patients (40–50) may be due to the fact that the size of this population was not enough to show the significance of the results. However, there are also other reasons why women with already-diagnosed fibroids should use hormonal contraception [7,8,9,10,11,12]. Uterine myomas have drawn much attention since being described more than 200 years ago. These benign uterine tumors often present with prolonged menstrual abnormalities (e.g., heavy, irregular and prolonged uterine bleeding), iron deficiency anemia, bulk symptoms (e.g., pelvic pressure/pain, obstructive symptoms) and fertility issues [7]. Approximately 40 percent of our group of patients suffered from uterine myomas. More than 25 percent of them had a positive family history regarding myomas. According to our study, patients using contraception with a positive family history were diagnosed with uterine myomas less often. The majority of them used contraceptive tablets for an average of four years. Furthermore, the results of this study indicate a significant relationship between the presence of uterine fibroids and coexisting gynecological diseases such as endometriosis and ovarian cysts. Indeed, primary oral hormonal contraceptives were approved by the Food and Drug Administration (FDA) for the treatment of menstrual disorders [13,14]. Combined estrogen/progestogen preparations are preferentially the monophasic drugs of choice for cyclic intakes. Additionally, extended regimens or constant management are progressively applied [15]. This improves either the effectiveness or the attractive convenience of such regimens [16].

Menstruation-associated dysmenorrhea is a relatively frequent event for woman with uterine fibroids. Today, dysmenorrheal hormonal contraceptive pills as monophasic estrogen/progestogen combinations are considered as the first choice to subside pain symptoms [17,18,19]. In case of an insufficient effect, a “long-cycle regimen” or constant usage is recommended [14].

From our research group, 27.4% of all the patients had a positive family history regarding uterine fibroids and more than half of them had uterine fibroids. It could mean that there is a correlation between the burdened family medical history of uterine fibroids and their actual occurrence [20]. In their research, Van Voorhis et al. also concluded that a maternal history of fibroids may be the biggest risk factor for the development of fibroids in a largely Caucasian population of women [21]. Coexisting gynecological diseases were stated in 24.6% of females, 54.1% of them were also diagnosed with uterine fibroids. It is a statistically significant relationship between the presence of uterine fibroids and coexisting gynecological diseases. In a study conducted by Wong et al. on a group of 772 patients—20% of them had endometrial polyps [22]. As we mentioned earlier, the causes of fibroids are hormonal disorders. Wong et al. reported that high testosterone with high E2 (estradiol) was associated with an elevated risk of incident fibroids in midlife women who never reported fibroids before baseline [23,24,25].

L.A. Wise et al. reported that, although the association between oral contraceptive (OC) use and uterine fibroids has been studied extensively, no clear patterns have emerged [19]. On the other hand, Ross et al. described the risk of fibroids as consistently decreasing with the increasing duration of oral contraceptive use; the risk of fibroids was reduced by some 31% in women who had used oral contraceptives for 10 years. Thus, there are studies that suggest an increased risk of uterine fibroids with contraception [26], a reduced risk [27] and also similar risk [28,29]. In two studies, researchers observed an increased risk (20–29%) among women who initiated OCs prior to 17 years of age compared with never users [26]. In our case, the mean age at which women began to use contraception was 21 years. Therefore, it raises the suspicion that the young age at which contraception is used may increase the risk of uterine fibroids. As it turns out, the duration of contraceptive use is also significant. In our study, the Chi–Pearson index indicated that the longer the patient uses oral contraception, the higher the risk of uterine fibroids is. Chiaffarina at al. stated that long-term contraception may decrease the risk of uterine fibroids [5]. They reported that the protective effect of long-term oral contraceptive use on the risk of myomas may have a biological explanation. The potential protective effect of long-term oral contraceptive use observed in this study supports the hypothesis that ovarian hormones influence the risk of uterine fibroids. Thus, any factor that reduces the exposure of the myometrium to estrogens or increases progesterone levels, such as oral contraceptive use, would tend to reduce the risk of the diseases [30]. Ying and others also reported that the long-term use of oral contraceptives increased the risk of uterine leiomyomas in women [31].

The results of the study show that taking the morning-after pill reduces the risk of uterine fibroids. The main ingredient of such a tablet is ulipristal acetate (UPA); it is an oral synthetic progesterone receptor modulator (SPRM) with high binding affinity for this receptor in humans. It has a tissue-specific and partially progesterone-antagonistic effect. In their research, Piecak et al. focused on the influence of SPRM on the development of uterine fibroids, stating that in vitro studies have shown that progesterone stimulates the proliferation of fibroid cells. These studies also demonstrated that SPRMs (asoprisnil, telapristone acetate and UPA) inhibit cell proliferation. This process induces apoptosis selectively in fibroid cells by downregulating antiapoptotic factors, antifibrotic activity and reducing or blocking growth factor expression, and the conclusion of the entire study was that UPA is the most effective pharmacological management of fibroids, and, in many cases, it may be an alternative to surgical treatment [32]. In their study, Biglia et al. describe the mechanism of action of the UPA as a progesterone antagonist, which inhibits the proliferation of leiomyoma cells and induces apoptosis by increasing cleaved caspase-3 expression and decreasing Bcl-2 expression. Moreover, UPA down regulates the expression of angiogenic growth factors, such as vascular endothelial growth factor (VEGF) and their receptors. Thus, it suppresses neo-vascularization, cell proliferation and survival in leiomyoma cells but not in normal myometrial cells [33]. Earlier studies in fibroid research demonstrated that cultured leiomyoma cells responded to 100 ng/mL of UPA (also known as CDB-2914; CDB-2914 (17α-acetoxy-11β-[4-*N*,*N*-dimethylaminophenyl]-19-norpregna-4,9-diene-3,20-dione) by regulating progesterone receptor (PR) isoforms [34]. Notably, the effect of UPA on the expression of PR-A was observed to be a dose-dependent increase in leiomyoma cells; a finding not appreciated in normal myometrial cells [35]. However, as the use of the morning-after pill is accidental, it may reduce the risk of the formation and growth of uterine fibroids. In light of the new data on the toxicity of ulipristal acetate and other new SPRMs still in clinical trials, their influence on uterine fibroids is still under investigation. Furthermore, Del Forno et al., in their study, look into liver function, tolerability and satisfaction during treatment with ulipristal acetate in women with fibroids. No cases of increased serum aspartate transaminase (AST) and alanin transaminase (ALT) levels were detected, and no woman reported symptoms suggestive of liver injury. The majority of women declared an improvement of fibroids-related symptoms and a high degree of satisfaction [36].

## 5. Conclusions

Contraceptive use decreases the probability of uterine fibroids, especially among patients between 30–40 years of age. The risk of myomas due to a positive family history can also be diminished after taking OC. Coexisting gynecological diseases, pregnancy and gynecological surgeries increases the risk of possible uterine fibroids.

### Limitations

This study contains particular limitations concerning the strength of the study and the control groups. The study did not cover the entire population but only the women who had the possibility to fill in the questionnaires (women enrolled during a hospital stay, women with internet access, women active on social media). A strong point of the study was the fact that there were no lacking answers, probably because of the easy and simple questions.

## Figures and Tables

**Table 1:** Characteristics of studied population.

Characteristics	Patients with Myomas (Study Group) *n* = 140 Mean ± SD	Patients without Myomas (Control Group) *n* = 206 Mean ± SD	*p* Value
Mean age (years)	36.61 ± 8.60	24.77 ± 6.17	*p* < 0.0001
Weight (kg)	67.61 ± 12.91	62.35 ± 12.48	*p* = 0.000004
Mean age of menarche (years)	12.99 SD = 2.08	12.74 SD = 1.59	*p* = 0.2298
Number of pregnancies (median)	0	1	*p* < 0.000001
Mean age of first pregnancy (years)	25.26 SD = 5.37	24.14 SD = 4.24	*p* = 0.0897
Median number of life births	1	1	*p* = 0.725
% of patients with natural labor (73 patients–100%)	63.01%	26.99%	
% of patients with cesarean labor (47 patients–100%)	63.83%	26.17%	

**Table 2:** Contraceptive use in the study and control group.

	Patients with Myomas (Study Group) *n* = 94	Patients without Myomas (Control Group) *n* = 180	*p* Value
Mean age [years]	23.71 SD = 7.00	19.63 SD = 4.04	*p* = 0.000000004
Mean duration of contraceptive use [months]	73.29 SD = 72.6	39.04 SD = 46.90	*p* = 0.00007

**Table 3:** Results of Chi2 test in the groups of patients divided according to age.

Age [years]	Myoma Presence	No Contraception Intake	Contraception Intake	Statistical Significance
20–30	Yes	9	15	*p* = 0.1159
	no	21	77	
30–40	yes	23	29	*p* = 0.00299
	no	3	25	
40–50	yes	14	26	*p* = 0.5422
	no	0	3	

**Table 4:** Relationships concerning uterine myomas.

Relationship between Uterine Fibroids			
	uterine fibroids present “+” lack of uterine fibroids “–”	statistical test used in analysis	*p* Value
Contraception	–	Chi2 Test	*p* = 0.00001
Morning-after pill taken	+	Chi2 Test	*p* = 0.67559
Positive family history	+	Chi2 Test	*p* = 0.00004
Gynecological surgeries	+	Chi2 Test	*p* < 0.00001
Gyn. coexisting diseases	+	Chi2 Test	*p* = 0.00275
Hypertension occurrence	+	Fisher’s exact two-sided test	*p* = 0.05593
Pregnancy number	–	U Mann–Whitney Test	*p* < 0.00001
Natural labor	–	Chi2 Test	*p* = 0.9278
Assisted labor	+	Chi2 Test	*p* = 0.1024
Age of first pregnancy	+	U Mann–Whitney Test	*p* = 0.0897

## Data Availability

By Authors upon request.
